# Scanning electron microscopy as a tool for evaluating morphology of amyloid structures formed on surface plasmon resonance chips

**DOI:** 10.1016/j.dib.2018.05.129

**Published:** 2018-05-26

**Authors:** Kristoffer Bra¨nnstro¨m, Anna L. Gharibyan, Tohidul Islam, Irina Iakovleva, Lina Nilsson, Cheng Choo Lee, Linda Sandblad, Annelie Pamren, Anders Olofsson

**Affiliations:** a Umeå University, Department of Medical Biochemistry and Biophysics, Linneaus väg 4, Umeå SE 90187, Sweden; b Umeå University, Umeå Core Facility for Electron Microscopy (UCEM), Linneaus väg 4, Umeå SE 90187, Sweden

## Abstract

We demonstrate the use of Scanning Electron microscopy (SEM) in combination with Surface Plasmon Resonance (SPR) to probe and verify the formation of amyloid and its morphology on an SPR chip. SPR is a technique that measures changes in the immobilized weight on the chip surface and is frequently used to probe the formation and biophysical properties of amyloid structures. In this context it is of interest to also monitor the morphology of the formed structures. The SPR chip surface is made of a layer of gold, which represent a suitable material for direct analysis of the surface using SEM. The standard SPR chip used here (CM5-chip, GE Healthcare, Uppsala, Sweden) can easily be disassembled and directly analyzed by SEM. In order to verify the formation of amyloid fibrils in our experimental conditions we analyzed also in-solution produced structures by using Transmission Electron Microscopy (TEM). For further details and experimental findings, please refer to the article published in Journal of Molecular Biology, (Brännström K. et al., 2018) [1].

**Specifications Table**

**Table:** 

Subject area	*Physics, biophysics*
More specific subject area	*Imaging*
Type of data	*Scanning electron microscopy (SEM), transmission electron microscopy(TEM).*
How data was acquired	*SEM data was acquired using a Zeiss Gemini, (GmbH, Germany) field emission microscope*
	*TEM data was acquired using a JEM1230 transmission electron microscope (JEOL, Watchmead UK)*
Data format	*Filtered*
Experimental factors	SEM analysis of Aβ fibrils grown on a CM5-chip (Uppsala, Sweden)TEM analysis of in-solution produced Aβ fibrils on a carbon coated copper grid
Experimental features	*SEM: A morphological analysis of Aβ fibrils grown on an SPR chip using SEM. Sonicated Aβ fibrils display a very short morphology. Probing fibrils with their monomeric counterpart facilitate growth through polymerization. The morphology of both sonicated (sheared) and non-sonicated fibrils are included as controls.*
	*TEM: A morphological analysis of mature Aβ-fibrils*
Data source location	*Dept. Medical biochemistry and Biophysics, Umeå University*
Data accessibility	*Data is included in this article*

**Value of the data**

• Combining SPR and SEM represents a valuable tool for monitoring the morphology of amyloid fibrils acquired as a result of a controlled polymerization.
• The technique facilitates morphological analysis followed by seeding and cross-seeding between different amyloids
• The technique may be expanded to other systems both within and outside the field of amyloid research.

## 1 Data

**Fig. 1:**
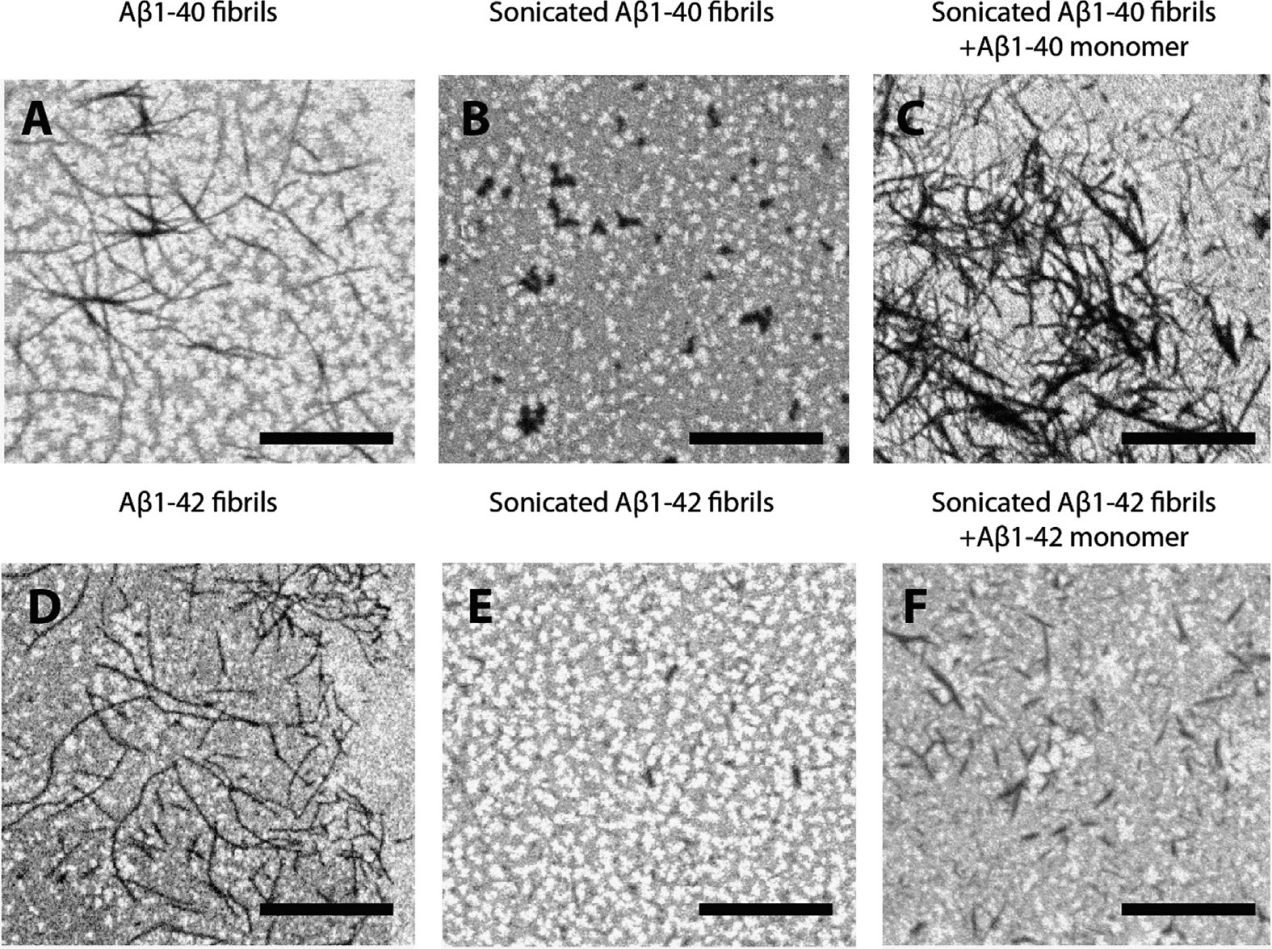
SEM analysis of the SPR chip surfaces exposes the morphology of the Aβ-assemblies. (A) Aβ_1–40_ fibrils prepared in PBS under stagnant incubation conditions followed by immobilization on the SPR chip surface. (B) Aβ_1–40_ fibrils sheared by microtip sonication to obtain very short fibrillar pieces and immobilized on the chip surface. (C) The sonicated and immobilized Aβ_1–40_ fibrils probed with monomeric Aβ_1–40_. (D) Aβ_1–42_ fibrils prepared in PBS under stagnant incubation conditions followed by immobilization on the SPR chip surface. (E) Aβ_1–42_ fibrils sheared by microtip sonication to obtain very short fibrillar pieces, and immobilized on the chip surface. (F) The sonicated and immobilized Aβ_1–42_ fibrils probed with monomeric Aβ_1–42_.

Fig. 1A and 1D shows SEM images of Aβ_1–40_ and Aβ_1–42_ fibrils prepared through prolonged incubation in phosphate-buffered saline (PBS) under stagnant conditions followed by immobilization on the CM5 chip [1]. The presence of the dextran surface generates a notable background in all SEM images. However, it does not impair visualization of the overall morphology of the aggregates. Although the resolution cannot match that of transmission electron microscopy (TEM) technique, the fibrillar morphology is clearly observed in both samples.

SPR is frequently used to probe the properties of amyloid formation [2], [3], [4], [5], [6], [7]. To further show that polymerization into fibrils actually occurs on the chip surface; the fibrillar morphology of the parental seeds first has to be altered in order to facilitate discrimination between the parental seeds and the potentially newly formed fibrils – while maintaining their molecular structure and templating ability. Both of these requirements could be achieved through sonication of the fibrils prior to immobilization, where the shearing forces generate very short fragments of the original molecular architecture of the fibril. Fig. 1B and 1E shows the sonicated Aβ_1–40_ and Aβ_1–42_ fibrils, respectively, where only short fibrillar fragments can be seen. This facilitates discrimination between the parental seeds, which are short, and the potentially new elongated fibrils formed as a result of the incorporation of monomers.

The immobilized and sonicated fibrils were then subjected to a prolonged exposure (200 min) of monomers at 0.5 µM in the SPR apparatus. Fig. 1C shows the sonicated Aβ_1–40_ fibrils exposed to monomeric Aβ_1–40_ and clearly shows the re-appearance of fibrillar structures. Similarly, Fig. 1F shows the sonicated Aβ_1–42_ fibrils after exposure to Aβ_1–42_ monomers, which results in a fibrillar morphology similar to the original morphology shown in Fig. 1C.

For all of the SPR experiments, a control surface was monitored to verify that no nonspecific binding, e.g. to the dextran surface, occurred. In this context, it is also important to emphasize that for all work involving Aβ, the samples were always subjected to size-exclusion chromatography immediately prior to use. This treatment effectively removes possible traces of aggregated material.

The combined use of SPR and SEM, where a morphological study of the surface of the SPR chip is performed by SEM, creates a powerful tool to correlate kinetic measurements and morphology. It should also be noted here that the signal from the SPR technique depends on the distance between the sample and the surface of the chip. The polymeric nature of an extending amyloid fibril means that the average distance to the surface will increase as the fibrils grow and that the response will eventually fall out of the linear range. To perform detailed kinetic measurements, the injection times and amounts of added mass should always be kept low to be within the linear range. However, to acquire a significant morphological change and be able to discriminate the newly formed fibrils from their templating parental fibrils, significantly prolonged incubation times are required and we have allowed 200 min for association to occur. As a consequence of the increased fibrillar length, the sensograms fall out of the linear range and are therefore not shown here.

## 2 Transmission electron microscopy (TEM) verified fibrillar morphology

**Fig. 2:**
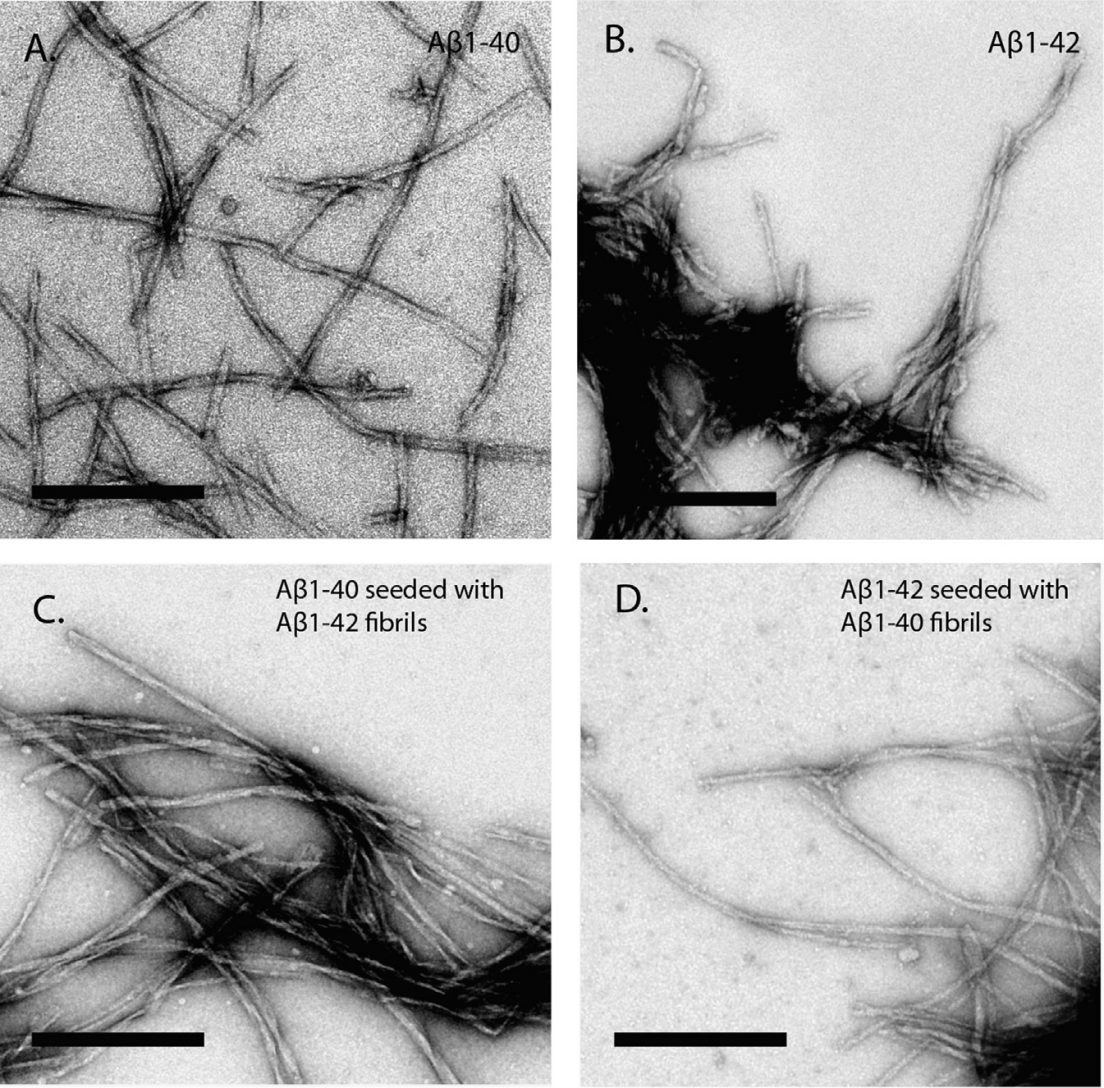
TEM analysis of fibrillar morphologies. (A) Fibrils of Aβ_1–40_ acquired through prolonged incubation in PBS under stagnant conditions. (B) Fibrils of Aβ_1–42_ acquired through prolonged incubation in PBS under stagnant conditions. (C) Fibrils of Aβ_1–40_ acquired through cross-seeding in PBS solution using Aβ_1–42_ fibrils as seeds under stagnant conditions. (D) Fibrils of Aβ_1–42_ acquired through cross-seeding in PBS solution using Aβ_1–40_ fibrils as seeds under stagnant conditions. The indicated scale bar in all images is 200 nm.

The use of thioflavin-T (ThT) is an established setup to monitor amyloid formation in solution. Although binding of ThT is indicative of amyloid structures, the presence of fibrillar morphology must be verified also using this method. Using TEM in combination with negative uranylactetate staining, the morphology of intrinsically formed Aβ_1–40_ and Aβ_1–42_ fibrils could be evaluated (Fig. 2A and B, respectively). In both cases, fibrillar morphology with a similar ultrastructure was revealed. The morphology displayed straight fibrils having a diameter around 10 nm and an indefinite length frequently exceeding several microns. Fig. 2C and 2D are representative images of Aβ_1–40_ and Aβ_1–42_ respectively, after cross-seeding via fibril-catalyzed secondary nucleation. The results confirm fibrillar morphology, but in analogy to the intrinsically formed fibrils no morphological differences are observed.

## 3 Experimental design, materials and methods

### 3.1 Scanning electron microscopy (SEM)

Because the CM5 chip used for SPR analysis has a gold surface that covers the sensor surface, the chips can be used directly to visualize the morphological structures of the fibrils. Before analysis, the PBS running buffer used with the CM5 chip for the SPR experiments was exchanged for distilled water to remove any traces of salt. The CM5 chip was subsequently dismantled and mounted onto an aluminum stub using carbon adhesive and copper tape. The fibril morphology was examined with field-emission SEM (Zeiss Gemini, GmbH, Germany) using an in-lens secondary electron detector at an accelerating voltage of 3 kV and a probe current of 90 pA.

### 3.2 Negative staining transmission electron microscopy (TEM)

Total volumes of 4 μL from the corresponding samples were adsorbed for 2 minutes onto glow-discharged carbon-coated copper grids, washed in water, and immediately negatively stained in 50 μL of 1.5% uranyl acetate solution for 30 seconds. Negative-stained samples were examined on a JEM1230 transmission electron microscope (JEOL) which was operated at 80 kV. Micrographs were recorded with a Gatan UltraScan 1000 2k × 2k pixel CCD camera using Digital Micrograph software.
